# Gypenosides attenuate lipopolysaccharide-induced neuroinflammation and anxiety-like behaviors in rats

**DOI:** 10.1080/19768354.2018.1517825

**Published:** 2018-09-05

**Authors:** Bombi Lee, Insop Shim, Hyejung Lee, Dae-Hyun Hahm

**Affiliations:** a Acupuncture and Meridian Science Research Center, College of Korean Medicine, Kyung Hee University, Seoul, Republic of Korea; b Center for Converging Humanities, Kyung Hee University, Seoul, Republic of Korea; c Department of Physiology, College of Medicine, Kyung Hee University, Seoul, Republic of Korea

**Keywords:** Anxiety, gypenosides, inflammation, lipopolysaccharide, nuclear factor-kappaB

## Abstract

Neuroinflammation is considered a major factor in several neuropsychiatric disorders. Gypenosides (GPS) have pharmacological properties with multiple beneficial effects including antiinflammatory, antioxidative, and protective properties. The present study was performed to examine whether GPS shows anxiolytic-like effects in a model of chronic inflammation induced by injection of lipopolysaccharide (LPS) into the rat hippocampus. The effects of GPS on inflammatory factors in the hippocampus and the downstream mechanisms of these effects were also examined. Introduction of LPS into the lateral ventricle caused inflammatory reactions and anxiety-like symptoms in the rats. Daily treatment with GPS (25, 50, and 100 mg/kg) for 21 consecutive days significantly increased the time spent and number of visits to the open arm in the elevated plus maze test, and significantly increased the number of central zone crossings in the open field test. Moreover, GPS administration significantly reduced the freezing response to contextual fear conditioning, and significantly decreased the levels of proinflammatory mediators, such as interleukin-1*β* (IL-1*β*), interleukin-6 (IL-6), and nuclear factor-kappaB (NF-κB), levels in the brain. Furthermore, GPS reduced LPS-induced elevated levels of Toll-like receptor 4 (TLR4) mRNA and inhibition of brain-derived neurotrophic factor (BDNF) mRNA levels. Taken together, these results suggest that GPS may have anxiolytic-like effects and may have novel therapeutic potential for anxiety-like behaviors caused by neuroinflammation. GPS may be useful for developing an agents for the treatment of neuropsychiatric disorders, such as anxiety, due to its antiinflammatory activities and the modulation of NF-κB/iNOS/TLR4/BDNF.

## Introduction

Anxiety disorders, also referred to as generalized social anxiety and panic disorders, are the most common mental health disorders, and are characterized by irritability, fatigue, restlessness, muscle tension, sleep problems, intense and persistent fear of social situations, and recurrent and unexpected panic attacks (Yang et al. 2017). Many recent studies have demonstrated that neuroinflammatory and sustained increases in various proinflammatory mediators, such as interleukin-1β (IL-1β), interleukin-6 (IL-6), and tumor necrosis factor-alpha (TNF-α), in the central nervous system are closely related to neuropsychiatric disorders, such as anxiety (Dantzer et al. 2008; Vogelzangs et al. 2013). Lipopolysaccharide (LPS) is a non-infectious element of the external membranes of gram-negative bacteria that regulates proinflammatory cytokines (Yang et al. 2017). Systemic injection of LPS causes physiological or psychiatric changes such as anhedonia, anorexia, depressed mood, apathy and anxiety (Dantzer et al. 2008). Therefore, LPS is used frequently in research on the biochemical mechanisms of anxiety-like behaviors due to inflammation and for the development of targeted therapies for neurological symptoms in animal models (Jin et al. 2017). LPS stimulates proinflammatory cytokine cascades via Toll-like receptor 4 (TLR4) and activation of the nuclear factor-kappaB (NF-κB) system (Fu et al. 2013). Nutraceuticals targeting neuroinflammatory mediators have been suggested as novel therapeutic tools for the management of neuropsychiatric and neuroinflammatory disorders (Sulakhiya et al. 2016).

Gypenosides (GPS) are compounds derived from *Gynostemma pentaphyllum*, the saponin extracts of which have long been used in Southeast Asia for the treatment of hyperlipidemia, cardiovascular disorders, and other chronic inflammation conditions (Shin et al. 2015). The active component of GPS is the hydroxyl group attached to carbon 20 or 21 of the dammarane-type ring (Shang et al. 2006). Using modern scientific methods, GPS has been shown to have a range of pharmacological properties, including neuroprotective, cardioprotective, antiaging, and antitumor effects (Megalli et al. 2005). For instance, GPS exerted antioxidative effects in primary cultures of cortical cells in glutamate-treated rats, and in the hippocampal CA1 region and cortex in a chronic cerebral hypoperfusion rat model (Shang et al. 2006; Zhang et al. 2011). In addition, GPS was shown to improve superoxide dismutase (SOD) activity in tissues and serum, increase antioxidant capabilities, and decrease levels of oxidized low-density lipoprotein (Li et al. 2017). In rats, GPS was shown to alleviate myocardial ischemia-reperfusion injury by preserving mitochondrial function and attenuating oxidative stress (Yu et al. 2016). Meanwhile, GPS exerted neuroprotective effects on 1-methyl-4-phenylpyridinium (MPP)^+^-induced dopaminergic neurons in primary nigral cultures (Wang et al. 2010) and improved the affective symptoms in a 1-methyl-4-phenyl-1,2,3,6-tetrahydrophyfidine (MPTP)-induced mouse model of Parkinson's disease (PD) (Shin et al. 2014). Moreover, GPS attenuated levodopa (_L_-DOPA)-induced dyskinesia in a 6-hydroxydopamine (6-OHDA)-induced PD rat model (Shin et al. 2014). These studies suggest that GPS may be useful for suppressing inflammation in psychiatric disorders.

Here, the anti-inflammatory efficacy of GPS on anxiety-like symptoms was investigated in a rat model of LPS-treated neuroinflammation as measured with the elevated plus maze (EPM) test, open field test (OFT), and contextual fear conditioning and fear extinction. We also examined how these effects were associated with the molecular modulation of neuroinflammation in terms of the neural mechanisms underlying the anxiolytic-like activity of GPS.

## Materials and methods

### Animals

Five-week-old adult male Sprague-Dawley rats (Samtako Animal Co., Seoul, Korea), weighing 195–210 g, were used in all experiments. The rats were housed in a limited access rodent facility with up to five rats per polycarbonate cage. The rats were sustained under a 12-h light/dark cycle (lights on at 08:00, lights off at 20:00) under controlled temperature (24°C ± 3°C) and relative humidity (50% ± 5%). All of the animals were allowed 7 days to adapt to these conditions after arrival at the facility. All of the methods were approved by the Animal Care and Use Committee of Kyung Hee University [KHUASP(SE)-15-115]. All of the procedures were performed in accordance with the Guide for the Care and Use of Laboratory Animals issued by the Korea National Institute of Health.

### Lesion generation and lipopolysaccharide administration

**Figure 1.**
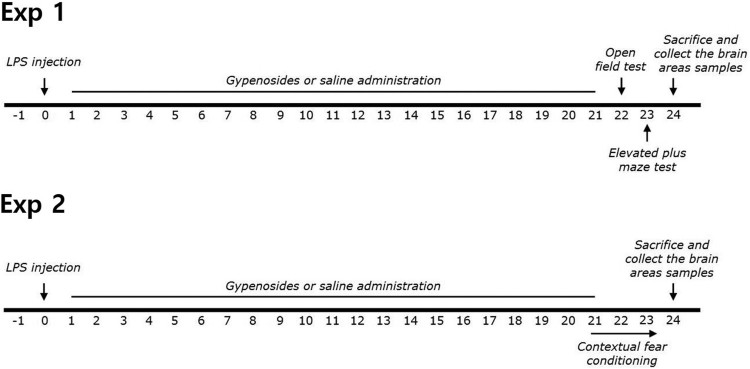
Experimental schedule of lesion generation, gypenosides administration, and behavioral tests in rats. LPS; lipopolysaccharide.

Gypenosides, ibuprofen (IBU), and LPS (Escherichia coli; O127:B8) were obtained from Sigma-Aldrich (St. Louis, MO, USA). LPS was administrated intracerebroventricularly (i.c.v.) into the lateral ventricle of the rat brain under anesthesia with intraperitoneal injection (i.p.) of 50 mg/kg sodium pentobarbital. LPS (50 μg) was dissolved in 10 μL of cerebrospinal fluid (CSF; Sigma). Injection of LPS was performed at a rate of 2 μL/min (total: 5 min) and the injection needles were left in place for an additional 5 min. The standard dose of LPS in rats and the long-term treatment schedule used in the present study were based on a previous study (Lee et al. 2013). Sham control animals were administered vehicle alone (i.c.v. and i.p.) instead of one of the drug solutions. GPS and IBU were dissolved in 0.9% saline before use. The entire experimental schedule of LPS injection and behavioral examinations is shown in Figure 1.

### Elevated plus maze test

The EPM test was carried out according to a previously described method (Lee et al. 2014). Briefly, rats were transferred to the EPM, which consisted of a four-armed wooden platform in the shape of a plus sign. The apparatus was painted with black enamel and was raised 50 cm above the floor. All arms were 50 cm in length, 10 cm in width, and joined in the center to create a 10 cm^2^ central platform. Two arms facing away from each other were protected, whereas the remaining two arms remained open. At the start of each experiment, the rat could move freely for 5 min. Video footage of these sessions was scored, and the ratio of the total times spent in the open and closed arms was used to measure anxiety.

### Open field test

The rats completed the OFT before the EPM. The OFT was performed according to a previously described method (Lee et al. 2016). The open field area consisted of an enclosed square area made of dark opaque acrylic glass (60 × 60 cm) surrounded by walls (30 cm in height). All of the locomotor activities (animal movements) were measured with a computerized video-tracking system using the S-MART program (Panlab Co., Barcelona, Spain). The locomotor activity was evaluated in terms of total distance traveled in the container, and exploratory activity was evaluated based on measurements of the total number of line crossings during 5 min. Painted white lines divided the area into 16 squares (15 × 15 cm each). The time spent and number of zone crossings in the central and peripheral zones were observed. The number of rearing events for each rat was also recorded to analyze locomotor activity in the OFT.

### Contextual fear conditioning and extinction

A separate group of rats that did not undergo EPM testing (*n* = 4∼5 per group) was tested for contextual fear conditioning and extinction after LPS injection. The contextual fear conditioning tests took place over 3 days following previously described procedures (Davies et al. 2016). Briefly, on the acquisition day, rats were placed in a foot shock chamber (30 cm long × 26 cm wide × 22 cm high) with an overhead camera, and were allowed to explore freely for 2 min. After the initial 2 min exploration period, a total of 10 electric shocks (0.75 mA, 2 s in duration) were delivered at intervals of 74 s through the testing chamber floor. The rats then remained in the chamber for an additional 5 min with no shocks delivered. On test days 1–2, rats were placed for 8 min in the same chambers as on acquisition day with no shocks delivered to determine the extent of contextual fear learning and extinction of contextual fear conditioning. This behavior was chosen because it was previously shown to induce re-experiencing of the aversive event and facilitate behavioral sensitization. The percentage of freezing responses was calculated by dividing the freezing time by the total time.

### IL-1β, IL-6, TNF-α, COX-2, and NF-κB measurement

Twenty-six days after LPS induction, IL-1β, IL-6, TNF-α, cyclooxygenase-2 (COX-2), and NF-κB concentrations in the hippocampus were assayed according to a previously described procedure (Lee et al. 2014). Three rats from each group were deeply anesthetized via isoflurane inhalation (1.2%), and were sacrificed one day after behavioral testing. The IL-1β, IL-6, TNF-α, COX-2, and NF-κB concentrations were evaluated with competitive enzyme-linked immunoassays (ELISAs) using anti-IL-1β antibody (Abcam, Cambridge, MA, USA), anti-IL-6 antibody (Abcam), anti-TNF-α antibody (Abcam), anti-COX-2 antibody (Abcam), and anti-NF-κB antibody (Abcam) according to the manufacturer's protocols.

### Total RNA isolation and reverse transcription-polymerase chain reaction

Inducible nitric oxide synthase (iNOS), TLR4, and brain-derived neurotrophic factor (BDNF) mRNA expression were evaluated by reverse transcription PCR according to a previously described method (Lee et al. 2014). Briefly, total RNA was extracted from the hippocampus of each rat using TRIzol reagent according to the manufacturer's instructions. cDNA was synthesized from 2 μg of RNA with PrimeScript reagent (Takara, Kyoto, Japan) on a thermal cycler (MJ Research, Watertown, MA, USA). Data were normalized relative to glyceraldehyde 3-phosphate dehydrogenase (GADPH) expression in the corresponding sample.

### Statistical analysis

All measurements were performed by an independent investigator blinded to the experimental conditions, and the results are expressed as the means ± standard error of the mean. Differences within or between normally distributed data were analyzed by analysis of variance (ANOVA) with SPSS (version 13.0; SPSS, Inc., Chicago, IL, USA) and Tukey's *post hoc* tests. Between subject two-way ANOVA was used to analyze the effects of GPS treatment and time. In all analyses, *p* < 0.05 was taken to indicate statistical significance.

## Results

### Effects of gypenosides on lipopolysaccharide-induced body weight loss

**Figure 2.**
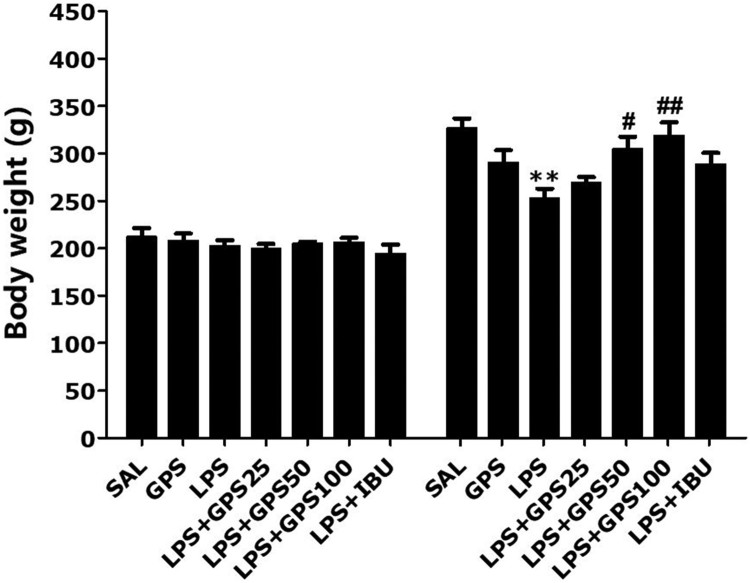
Effects of gypenosides on body weight gain on the first day before lipopolysaccharide injection and on day 21 after lipopolysaccharide injection. **p < 0.01 *vs*. SAL group; ^#^*p* < 0.05, ^##^*p* < 0.01 *vs*. LPS group.

We measured the body weight of each rat in each group on days 1 and 21 (Figure 2). The rats in the LPS group showed body weight loss during the 21 days of treatment relative to the saline-treated (SAL) group (*p* < 0.01). Interestingly, rats treated with GPS showed significant inhibition of the reduction in body weight gain compared to rats in the LPS group during the 21 days of treatment (50 mg/kg GPS, *p* < 0.05; 100 mg/kg GPS, *p* < 0.01), indicating that recovery of body weight in the GPS-treated groups was closely associated with that in the SAL group.

### Effects of gypenosides on lipopolysaccharide-induced anxiety-like behaviors

**Figure 3.**
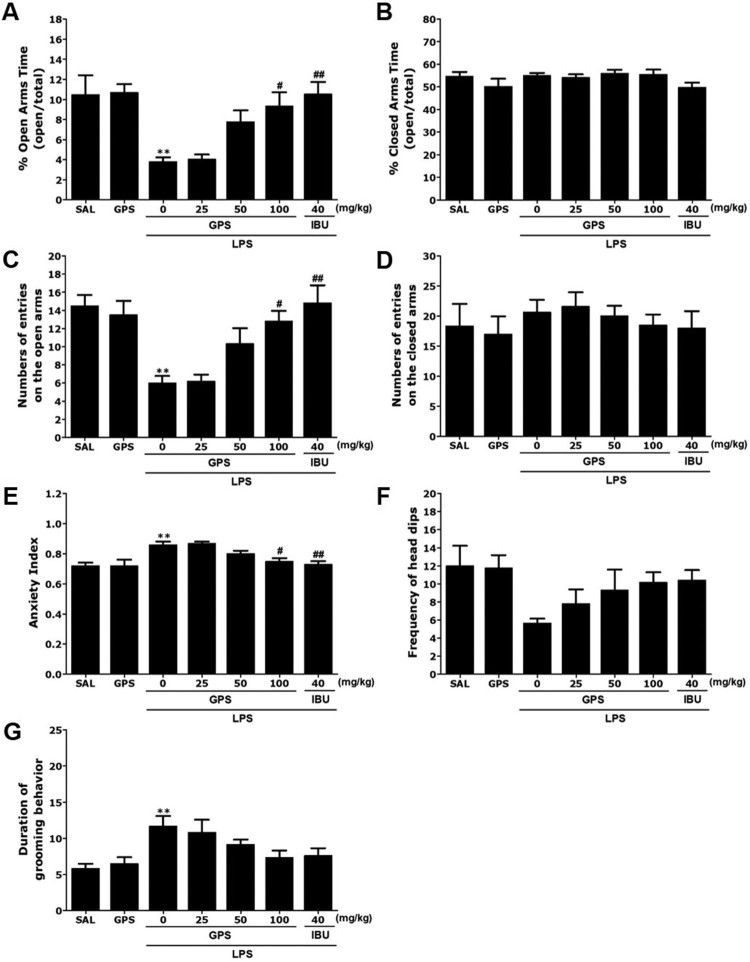
Effects of gypenosides administration on the percentage of time spent in the open and closed arms, numbers of entries into the open and closed arms, anxiety index, number of unprotected head dips, and grooming behavior in the elevated plus maze test after lipopolysaccharide injection. **p < 0.01 *vs*. SAL group; ^#^*p* < 0.05, ^##^*p* < 0.01 *vs*. LPS group.

Rats exhibited an obvious anxiety phenotype characterized by reduced open-arm exploration during the EPM test (Figure 3). *Post-hoc* comparisons indicated that the percentage of time spent and the number of entries into the open arms of the maze were significantly decreased in the LPS group compared with the control group (*p* < 0.01). However, rats in the LPS + GPS100 group showed significant restoration of the time spent and the number of entries into the open arms of the maze compared with the LPS group (*p* < 0.05). The percentage of time spent and the number of entries into the closed arms of the maze did not differ significantly among the seven groups [F(6,37) = 1.556, *p* = 0.193 and F(6.37) = 0.516, *p* = 0.791]. Overall, the anxiety index, calculated based on the number of visits to and time spent in the open and closed arms, also differed among the seven groups, with lower values in the GPS-treated rats (*p* < 0.05). Administration of GPS significantly increased the frequency of unprotected head dips compared with the LPS group, although this result was only marginally significant. However, the duration of grooming behavior was reversed by 100 mg/kg GPS when administered after LSP injection, although this result was only marginally significant.

### Effects of gypenosides on lipopolysaccharide-induced locomotion and exploratory behaviors

**Figure 4.**
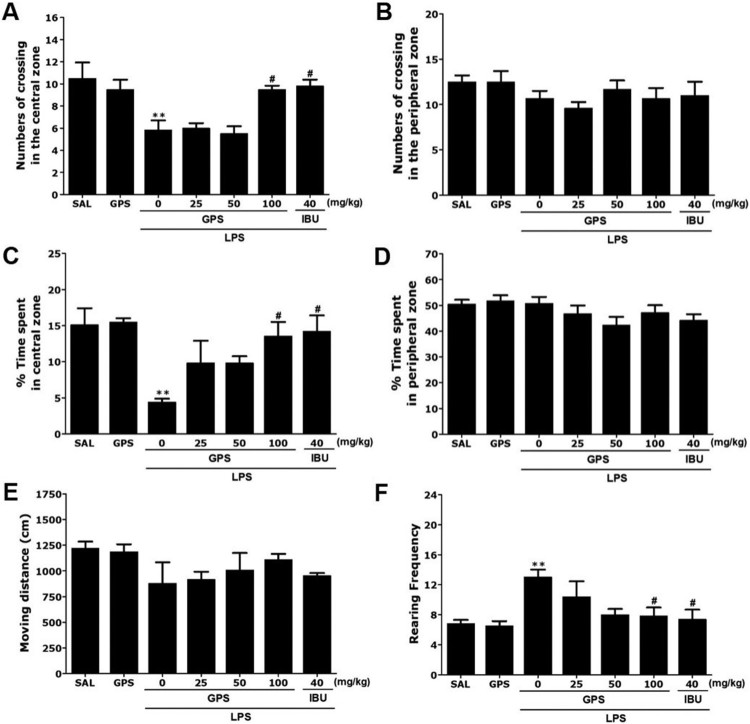
Effects of gypenosides administration on locomotion and exploratory behavior in the open filed test in rats exposed to lipopolysaccharide. Changes in the time spent in the central and peripheral zones, numbers of crossing in the central and peripheral zones, locomotor activity, and number of rearing events in the open field test after LPS injection. **p < 0.01 *vs*. SAL group; ^#^*p* < 0.05 *vs*. LPS group.

Rats exposed to LPS spent significantly less time in the central zone and correspondingly more time in the peripheral zone compared to the saline (SAL) group (*p* < 0.01; Figure 4). There was also a significant decrease in the number of central zone crossings following LPS injection (*p* < 0.01). Our results indicated that LPS-treated rats developed exploratory activities that were closely associated with the anxiety-like behaviors observed in the OFT. However, rats treated with 100 mg/kg of GPS showed significant increases in the time spent and number of central zone crossings compared with the LPS group (*p* < 0.05). Significant differences in the total number of rearing events (exploratory activities) were observed between saline-treated rats, the LPS group, and the GPS-treated groups [F(6,37) = 4.280, *p* < 0.01], but there were no significant differences in locomotor activity (motor function) between the groups [F(6,37) = 1.193, *p* = 0.336]. These results indicated that there were no effects on locomotor activities in the OFT in any of the groups. Rats exposed to LPS showed significantly increased frequency of rearing events in the open field compared with the SAL group (*p* < 0.01). However, rats in the LPS + GPS100 group showed significant restoration of raring frequency in the open field compared with in the LPS group (*p* < 0.05).

### Effects of gypenosides on lipopolysaccharide-induced contextual fear conditioning and extinction

**Figure 5.**
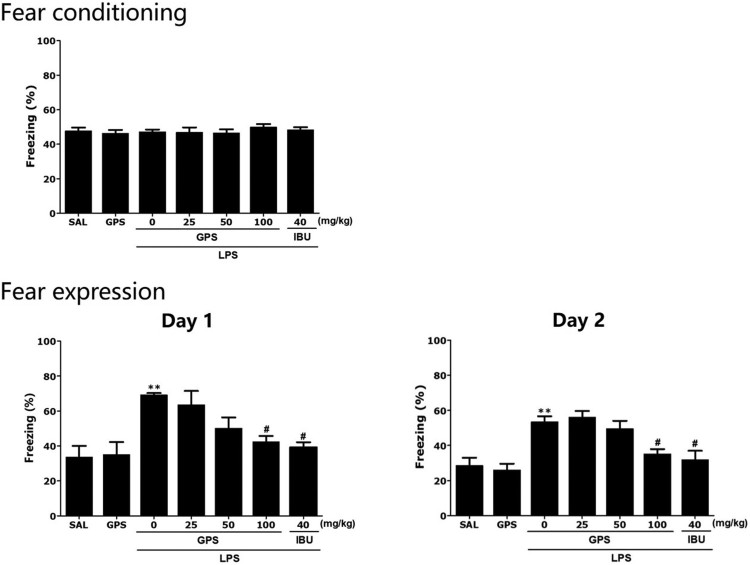
Effects of gypenosides on freezing behavior from contextual fear conditioning testing after lipopolysaccharide injection. The percentage of time spent freezing was determined on acquisition, day 1, and day 2. **p < 0.01 *vs*. SAL group; ^#^*p* < 0.05 *vs*. LPS group.

The effects of GPS administration on contextual freezing behavior of rats are shown in Figure 5. Unconditioned freezing duration in response to foot shock did not differ between groups [F(6,37) = 0.145, *p* = 0.347]. In contrast, two way ANOVA across the three testing sessions revealed a significant main effect of treatment [F(6,37) = 9.45, *p* < 0.01], a significant main effect of time [F(6,37) = 29.51, *p* = 0.145] and a significant interaction between treatment and time [F(6,37) = 2.412, *p* < 0.05] for freezing behavior. In the contextual freezing measurement, the freezing time increased significantly after injection of LPS (*p* < 0.01). The percentage of time spent displaying freezing behavior was significantly decreased in the group treated with 100 mg/kg GPS on days 1 and 2, respectively (*p* < 0.05). These results indicated that a persistent fear response to the original context was associated with the traumatic events that occurred there and that repeated GPS treatment ameliorated the context-dependent freezing behavior in rats.

### Effects of gypenosides on lipopolysaccharide-induced changes in inflammatory mediators in the hippocampus

**Figure 6.**
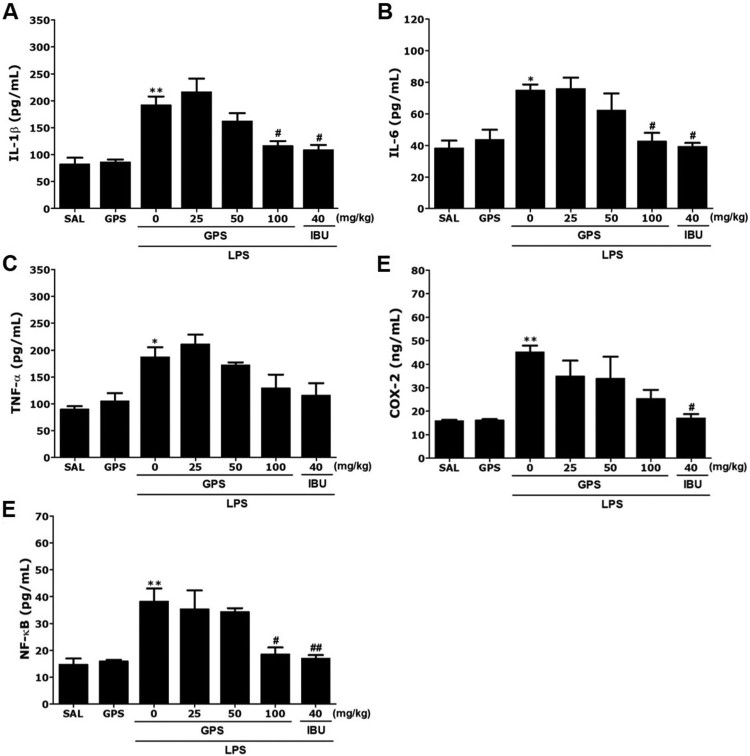
Effects of gypenosides on interleukin-1*β* (IL-1*β*)(A), interleukin-6 (IL-6)(B), tumor necrosis factor-α (TNF-α)(C), cyclooxygenase-2 (COX-2)(D), and nuclear factor-kappaB (NF-κB)(E) concentrations in the brains after lipopolysaccharide injection. *p < 0.05, **p < 0.01 *vs*. SAL group; ^#^*p* < 0.05, ^##^*p* < 0.01 *vs*. LPS group.

Following the behavioral tasks, brain tissue samples from the rats were analyzed to investigate the effects of GPS administration on the expression of proinflammatory markers activated by LPS-induced inflammation in the hippocampus (Figure 6). The hippocampal levels of IL-1β, IL-6, TNF-α, COX-2, and NF-κB showed significant differences between groups. The *post-hoc* test indicated significant increases in IL-1β levels in the hippocampus of the LPS group compared with the groups not treated with LPS (*p* < 0.01)(Figure 6(A)). Daily administration of GPS significantly decreased the LPS-induced increase in IL-1β concentration in the hippocampus compared with the LPS group (*p* < 0.05). After GPS treatment, the level of IL-6 in the hippocampus was also significantly decreased to 56.82% of that in the LPS group (*p* < 0.05)(Figure 6(B)).

ELISA showed that LPS injection for 21 days significantly increased TNF-α and COX-2 concentrations in the hippocampus of rats by 208.06% and 280.87%, respectively, compared with the saline-treated controls (*p* < 0.05 and *p* < 0.01, respectively)(Figure 6(C and D)). However, administration of GPS inhibited the LPS-induced increase in TNF-α expression in the hippocampus, although this result was only marginally significant. Daily administration of GPS significantly decreased the LPS-induced increase in COX-2 concentration in the hippocampus compared with the LPS group (*p* < 0.05).

The *post-hoc* test results also indicated a significant increase in NF-κB levels in the hippocampus of the LPS groups compared with the SAL group (*p* < 0.01)(Figure 6(E)). Daily administration of GPS significantly decreased the LPS-induced increase in NF-κB concentration in the hippocampus compared with the LPS group (*p* < 0.05).

### Effects of gypenosides on lipopolysaccharide-induced expression of iNOS, TLR4, and BDNF mRNA in the hippocampus

**Figure 7.**
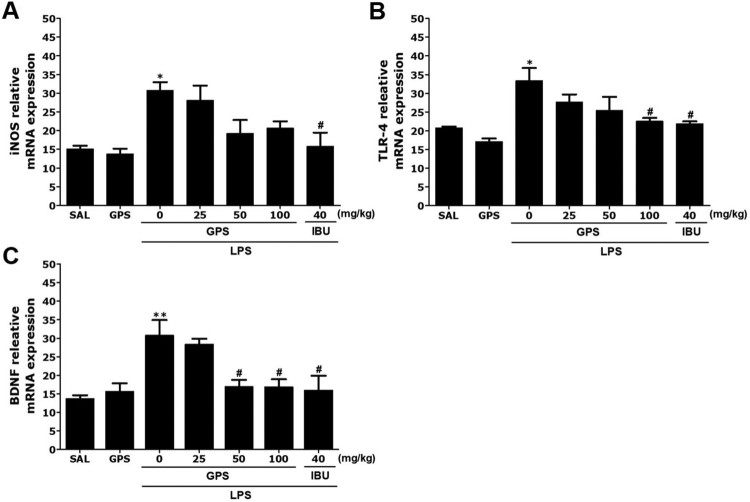
Effects of gypenosides on the expression of inducible nitric oxide synthase (iNOS)(A), Toll-like receptor 4 (TLR4)(B), and brain-derived neurotrophic factor (BDNF)(C) mRNAs in rats with lipopolysaccharide-induced hippocampal impairment. The expression levels of iNOS, TLR4, and BDNF mRNAs were normalized relative to glyceraldehyde 3-phosphate dehydrogenase (GAPDH) mRNA as an internal control. *p < 0.05 and **p < 0.01 *vs*. SAL group; ^#^*p* < 0.05 *vs*. LPS group.

Next, we analyzed the effects of GPS on the expression levels of iNOS, TLR4, and BDNF mRNA in the hippocampus of rats exposed to LPS (Figure 7). The mRNA levels of iNOS and TLR4 in the LPS group were significantly elevated compared with the SAL group (*p* < 0.05)(Figure 7(A)). The increased expression levels of iNOS mRNA in the LPS group were re-established to levels similar to those in the SAL group after administration of 100 mg/kg GPS, although this result was only marginally significant. The increased level of TLR4 mRNA expression in the LPS group was significantly re-established to a level similar to that seen in the SAL group after treatment with 100 mg/kg GPS (*p* < 0.05) (Figure 7(B)). In addition, BDNF mRNA levels in the LPS group were significantly decreased compared with those in the SAL group (*p* < 0.01) (Figure 7(C)). The decreased level of BDNF mRNA expression in the LPS group was significantly re-established to a level similar to that seen in the SAL group after treatment with 100 mg/kg GPS (*p* < 0.05).

## Discussion

The results of the present study clearly indicated that GPS treatment significantly increased the time spent in and the number of entries into the open arms in the EPM and reduced the anxiety index. Administration of GPS after LPS injection also significantly reduced the anxiety-like behaviors, as indicated by the increased number of central zone crossings in the OFT. GPS administration significantly enhanced the freezing response to contextual fear conditioning. In addition, this anxiolytic effect was accompanied by normalization of LPS-induced inflammatory mediators in the brain, as well as iNOS, TLR4, and COX-2 expression via modulation of NF-*κ*B activation in the brain. Administration of GPS also produced an increase in BDNF mRNA levels in the hippocampus associated with LPS-induced anxiety-like symptoms in experimental rats. Therefore, GPS dose-dependently mitigated LPS-induced anxiety-like behaviors via appropriate modulation of NF-*κ*B/TLR4/BDNF.

One study showed that intracerebroventricular administration of 2 μg of LPS induced memory impairment in the Morris water maze test one day after LPS injection (Zhang et al. 2015). However, another study indicated that a single injection of LPS (5 μg/5 uL) into the substantia nigra triggered an inflammatory response characterized by activation of glial cells and proinflammatory cytokine production, leading to behavioral abnormalities 21 days after LPS injection in rats (Sharma et al. 2017). Moreover, a single injection of LPS into the lateral ventricle of the rat brain induced production of proinflammatory cytokines, such as IL-1α, IL-1β, TNF-α, IL-6, and COX-2, as well as in mouse hippocampus for 3 weeks (Guo et al. 2010; Miwa et al. 2011). In the present study, we used long-term administration and the same dose of GPS administration as determined in previous studies (Zhang et al. 2011; Zhao et al. 2017). For example, to verify the protective effect of GPS on MPTP-induced neurotoxicity, the animals received GPS at 50 mg/kg (i.p., once per day) 1 h before MPTP injection for 21 days. Here, we expected both protective and therapeutic effects of GPS. Therefore, we chose a protocol of long-term GPS administration for 14 days after the LPS procedure in the present study.

The results of our behavioral investigations indicated anxiolytic-like effects of GPS in an animal model of anxiety. Administration of GPS after LPS injection significantly reduced anxiety-like behaviors in the EPM test, as indicated by increased exploratory behaviors, and more entries into the open arms (Yang et al. 2017). Administration of GPS after LPS injection also significantly increased the number of central zone crossings in the OFT (Yang et al. 2017). Therefore, because behaviors in the EPM test and OFT are related to LPS-related psychological symptoms, our results suggest that GPS may inhibit neuroinflammation.

Furthermore, consistent with previous studies, the results presented here indicated that LPS injection enhanced contextual freezing and anxiety-like behavior in the OFT, as well as LPS-induced analgesia compared with that the control group (Shin et al. 2014). LPS administration appears to impair contextual fear conditioning by disruption of the trial processes that consolidate a memory representation of the context (Pugh et al. 1998). Thus, the present results support the suggestion that GPS could ameliorate fear memory and anxiety induced by neuroinflammation.

Some studies have shown that LPS-induced inflammation impairs the contextual fear memory, as evidenced by the significantly decreased freezing time in response to the context. (Wang et al. 2016; Zhang et al. 2016). However, another study indicated that LPS injection decelerated extinction learning and potentially increased fear retention in contextual fear conditioning and extinction (Doenni et al. 2017). In addition, LPS-treated mice showed higher baseline freezing scores compared with vehicle-treated mice during the training phase (Avramescu et al. 2016). These results showed that LPS alone impaired both contextual and cued fear memory. In this study, these behavioral changes in rats after exposure to LPS through various behavioral tests and several anxiety indexes, such as freezing in contextual fear conditioning and extinction, suggested that anxiety-like behavior was successfully established. Therefore, our results showed that GPS treatment could alleviate anxiety-like behavior in LPS-treated rats, as indicated by their decreased freezing time in contextual fear conditioning and extinction.

In addition, our study indicated that infiltration of LPS into the lateral ventricle resulted in significant increases in IL-1β, IL-6, and TNF-α expression levels in the hippocampus, ultimately leading to a chronic neuroinflammatory response in the brain. LPS-stimulated sustained increases in the expression of proinflammatory cytokines have been directly linked to psychiatric disorders associated with anxiety-like behaviors (Sulakhiya et al. 2015). GPS continuously decreased LPS-induced IL-1β and IL-6 levels, ultimately resulting in improvement of persistent brain dysfunction and chronic inflammation (Sulakhiya et al. 2016).

Our results indicated that the inflammatory reactions to LPS infiltration significantly upregulated COX-2 mRNA and protein levels in the hippocampus by modulating the NF-*κ*B pathway (Gong et al. 2011). Increases in COX-2 levels due to NF-κB activation can accelerate inflammatory responses and subsequently contribute to anxiety-like behaviors (Gong et al. 2011). Thus, treatment with long-lasting COX-2 inhibitors during anxiety-like symptoms, before the appearance of clinical symptoms of common mental health disorders, may suppress inflammatory responses and the synthesis of proinflammatory mediators in the brain (Kumar et al. 2006). In the present study, GPS slightly decreased LPS-stimulated behavioral changes and anxiety-like symptoms by inhibiting the increases in COX-2 levels, although this effect was only marginally significant.

Furthermore, expression of inflammatory genes, such as iNOS and COX, and activation of NF-κB were inhibited by GPS treatment. Therefore, we speculate that GPS may prevent the deleterious effects of LPS on anxiety-like behaviors. In addition, our results may help to explain the association of GPS with intracellular NF-*κ*B, which is a major transcription factor that regulates genes responsible for both innate and adaptive immune responses (Fu et al. 2013). We found that the levels of multiple neuroinflammation markers induced by LPS via NF-*κ*B activation, including the LPS receptor TLR4, proinflammatory cytokines, and iNOS implicated in activation of NF-*κ*B and production of inflammation mediators, were reduced in GPS-treated rats compared with controls (Palsson-McDermott and O’Neill 2004). Our results demonstrated that GPS could mitigate the induction of proinflammatory mediators, including IL-1β and IL-6, and iNOS expression caused by LPS. In addition, GPS attenuated LPS-induced inflammatory damage by regulating TLR4/NF-*κ*B responsible for its neuroprotective effects.

We also investigated the levels of BDNF, an important factor associated with the pathogenesis of anxiety-like behaviors (Hritcu and Gorgan 2014). Because BDNF has critical roles in anxiety-like symptoms and hippocampal synaptic plasticity, maintenance of BDNF signaling is likely to contribute to the beneficial effects of GPS on anxiety-like symptoms in LPS-injected rats. In the present study, GPS treatment significantly reversed LPS-induced decreases in BDNF mRNA expression, indicating that the beneficial effects of GPS were mediated by increased BDNF mRNA expression, which is potentially associated with enhanced neuronal function and performance in anxiety-related tasks.

In summary, administration of GPS after LPS injection significantly increased the time spent exploring the open arms in the EPM test and reduced the anxiety index. Administration of GPS after LPS injection also significantly increased the number of central zone crossings in the OFT. Therefore, GPS was associated with anxiolytic-like effects in the OFT and EPM tests. GPS also suppressed LPS-simulated expression of proinflammatory mediators, such as IL-1β and IL-6, in the hippocampus via modulation of NF-*κ*B/iNOS/TLR4. Our results suggest that inflammation may cause anxiety-like behaviors by decreasing BDNF gene expression. Taken together, our results indicate that reductions in neuroinflammation may be crucial for preventing anxiety-like symptoms and that GPS may represent a useful therapeutic intervention.
